# Scaling Effect of Fused ASTER-MODIS Land Surface Temperature in an Urban Environment

**DOI:** 10.3390/s18114058

**Published:** 2018-11-20

**Authors:** Hua Liu, Qihao Weng

**Affiliations:** 1 Department of Political Science and Geography, Old Dominion University, Norfolk, VA 23529, USA; hxliu@odu.edu; 2 Center for Urban and Environmental Change, Department of Earth and Environmental Systems, Indiana State University, Terre Haute, IN 47809, USA

**Keywords:** land surface temperature, spatio-temporal image fusion, STARFM, downscaling, urban areas

## Abstract

There is limited research in land surface temperatures (LST) simulation using image fusion techniques, especially studies addressing the downscaling effect of LST image fusion. LST simulation and associated downscaling effect can potentially benefit the thermal studies requiring both high spatial and temporal resolutions. This study simulated LSTs based on observed Terra Advanced Spaceborne Thermal Emission and Reflection Radiometer (ASTER) and Terra Moderate Resolution Imaging Spectroradiometer (MODIS) LST imagery with Spatial and Temporal Adaptive Reflectance Fusion Model, and investigated the downscaling effect of LST image fusion at 15, 30, 60, 90, 120, 250, 500, and 1000 m spatial resolutions. The study area partially covered the City of Los Angeles, California, USA, and surrounding areas. The reference images (observed ASTER and MODIS LST imagery) were acquired on 04/03/2007 and 07/01/2007, with simulated LSTs produced for 4/28/2007. Three image resampling methods (Cubic Convolution, Bilinear Interpolation, and Nearest Neighbor) were used during the downscaling and upscaling processes, and the resulting LST simulations were compared. Results indicated that the observed ASTER LST and simulated ASTER LST images (date 04/28/2007, spatial resolution 90 m) had high agreement in terms of spatial variations and basic statistics based on a comparison between the observed and simulated ASTER LST maps. Urban developed lands possessed higher LSTs with lighter tones and mountainous areas showed dark tones with lower LSTs. The Cubic Convolution and Bilinear Interpolation resampling methods yielded better results over Nearest Neighbor resampling method across the scales from 15 to 1000 m. The simulated LSTs with image fusion can be used as valuable inputs in heat related studies that require frequent LST measurements with fine spatial resolutions, e.g., seasonal movements of urban heat islands, monthly energy budget assessment, and temperature-driven epidemiology. The observation of scale-independency of the proposed image fusion method can facilitate with image selections of LST studies at various locations.

## 1. Introduction

Land surface temperature (LST) is a primary factor of land-atmosphere energy exchange and is an important variable of urban thermal behavior and dynamics [1]. Thermal infrared satellite imagery is an efficient source of LST retrieval and numerous algorithms have been developed based on satellite and airborne sensors, e.g., Landsat Enhanced Thematic Mapper Plus (Landsat ETM+), Advanced Spaceborne Thermal Emission and Reflection Radiometer (ASTER), Advanced Very High Resolution Radiometer (AVHRR), and Heat Capacity Mapping Mission (HCMM). LST has been widely used to investigate heat-related phenomena and issues, e.g., urban heat islands [2], surface energy fluxes [3], and epidemiology of virus infections [4]. The LST patterns and their relationships with surface biophysical characteristics, e.g., vegetation, impervious surface, and human behavior have been well addressed in the literature [2,5,6]. LST is shown to be associated terrain topography (elevation and slope), e.g., LST tends to increase with more solar insolation at higher land; winds at steeper slopes may influence LST [7,8]. 

Image fusion is a digital technique to generate a dataset based on two or more observed images from different sources [9]. It has been commonly used to form high-spatial and high-temporal resolutions by integrating high-resolution panchromatic image and low-resolution multispectral image [10,11]. There are some popular image fusion approaches: (1) Intensity-Hue-Saturation (IHS) method, which transfers a multi-band image from Red Green Blue (RGB) to IHS mode and creates an IHS fused new image [12]; (2) Principal Component Analysis (PCA) that converts correlated multispectral bands into uncorrelated components and generates fused panchromatic image with high resolution [13];(3) arithmetic algorithms, e.g., Brovey Transform integrating multispectral bands and high-resolution panchromatic channel with a set of multiplication and division operations [14]; (4) wavelet approach, which links high-resolution panchromatic data with low-resolution multispectral band based on a reverse wavelet conversion with specific wavelet coefficients [15]; and (5) statistics-based fusion that applies statistical approaches to assess the relationship among input spectral bands and evaluate the influences of individual bands to the final fused image [16,17,18,19]. The Spatial and Temporal Adaptive Reflectance Fusion Model (STARFM) is an original and typical example of statistics-based fusion algorithm that simulates shortwave surface reflectance images based on observed Landsat and Moderate Resolution Imaging Spectroradiometer (MODIS) surface reflectance images [16]. There is another model called Spatio-Temporal Adaptive Data Fusion Algorithm for Temperature mapping (SADFAT) which is an improved version of STARFM for predicting thermal radiance and LST data by considering annual temperature cycle (ATC) and urban thermal landscape heterogeneity [17]. The Spatial Temporal Adaptive Algorithm for mapping Reflectance Change model (STAARCH) was designed to detect spatial and temporal changes in the landscape with great details of disturbance [18]. The Enhanced Spatial and Temporal Adaptive Reflectance Fusion Model (ESTARFM) was developed to improve the fusion accuracy on complex and heterogeneous landscapes compared to that of STARFM [19]. 

Scale influences the examinations of the landscape patterns and thermal behaviors on the earth’s surface. The scaling issue not only has a long history in various disciplines in general, e.g., biogeography, climatology, hydrology, and geomorphology [20,21,22,23], but also attracts wide attention in remote sensing and landscape studies specifically [24,25,26,27,28]. Schmid [29] observed that the thermal radiance could be stable across a range of scales (25–200 m) in urban areas with homogeneous land use land cover and LSTs. It was also believed that the thermal characteristics captured by a specific thermal inferred sensor may not necessarily be the same as the ones retrieved at different times with the same sensor, or the ones from other sensors [30]. Liu and Weng [31] suggested an optimal scale (90 m) in studying the relationship between LST and landscape pattern for a specific study site (the City of Indianapolis, USA) based on landscape metrics analysis. 

A challenge in remote sensing analysis is to obtain/generate a remote sensing image with both high spatial and high temporal resolutions. The restriction lies in the technical difficulties for any sensor to provide measurements, e.g., LST data of global coverage at a reasonably high spatial resolution while maintaining a high temporal resolution [17]. Image downscaling is one of the possible solutions to overcome the restriction. Different studies have been conducted to demonstrate the downscaling techniques in remote sensing, e.g., spectral mixture analysis, regression, cokriging, and Hopfield neural network [32,33,34]. Although diverse fusion methods have been successfully developed, most applications of image fusion have been focused on surface reflectance fusions. Relatively fewer documents address the possible applications of image fusion techniques in LST fusion which will potentially benefit the thermal studies requiring high spatial and temporal resolutions [35]. Zakšek and Oštir [36] applied a LST downscaling approach (principal component analysis and regression) for urban heat island assessment based on MODIS LST level 2 data. Another LST downscaling study included multispectral data and morphological conditions as downscaling predictors [37]. Jiang and Weng [38] examined the surface moisture based on downscaled LST over urban terrains using Zakšek’s method. Stathopoulou and Cartalis [39] used different scaling techniques to downscale AVHRR LST imagery and found that the downscaled LST imagery possessed visual improvement compared to that of the original data. Liu and Pu [40] estimated subpixel thermal infrared radiance by applying both physical and statistical downscaling models, and suggested that both downscaling models were suitable for maintaining the general patterns in the original image with considerable spatial details. An applied study assessed heat wave health risks based on downscaled Geostationary Operational Environmental Satellite (GOES) LST [41], and found that the downscaling method used (Zakšek’s algorithm [36]) could be used to effectively address the spatial and temporal variability of heat waves in urban areas. Various disaggregation methods were used to improve the spatial resolution of thermal infrared data based on Landsat visible and near infrared (VNIR) data and MODIS LST imagery, among which a linear regression method reached the best results [42]. Although the downscaling techniques on LSTs have been documented, there are very limited studies discussing the possible downscaling effects during the LST fusion process mentioned above, which can be critical in some thermal landscape issues, e.g., the central locations and magnitudes of urban heat islands in urban areas with various sizes [43], and the relationships between LSTs and landscape patterns [31]. 

The objectives of this study were: (1) to simulate land surface temperatures (LSTs) in an urban environment with an existing statistics-based image fusion model; and (2) to assess the downscaling effect in LST image fusion. The simulated LST images can be used to evaluate thermal landscape, energy exchanges, and other related phenomena that need LST information at a more frequent base with fine spatial resolutions, e.g., seasonal movements of urban heat islands, monthly energy budget assessment, and temperature-driven epidemiology. The downscaling effect analysis will facilitate with image selections of LST studies at various locations. 

## 2. Materials and Methods

### 2.1. Study Area

**Figure 1:**
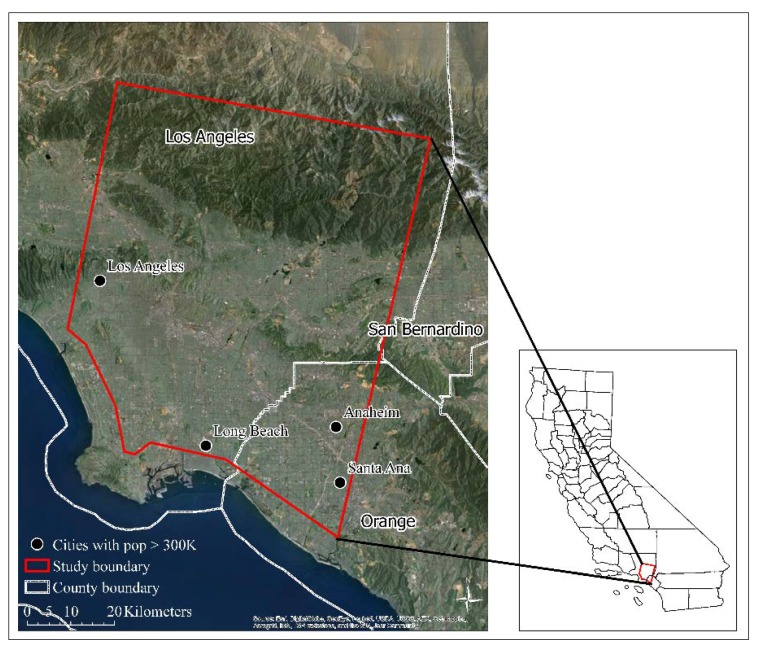
Geographical location of the study area.

The study area covered part of the City of Los Angeles (LA), California, USA and surrounding locations, e.g., Long Beach, Anaheim, and Santa Ana (Figure 1). The overall elevation increases from flat coastal land on the south to hilly mountains to the north with a range from about 5 m to 2590 m. Some hills are present in the central west and central east. Sitting along the coast, Los Angeles has a typical Mediterranean climate condition (e.g., hot and dry summers, and warm and moist winters). The average high temperature is 29.3 °C in August and 20.1 °C in January, according to the weather station on the Downtown-University of Southern California campus. Temperature transitions between the inland and coastal areas can be obvious and are closely related to elevation and distance from the coast. More than 60% of the area is covered by urban development mainly spreading along the coast and in the south with flatter land. Vegetation, e.g., shrubs and canopies mainly appear in residential areas, rural mountains on the north, and vacant fields. Certain bare soil and herbaceous vegetation can be observed as well [44]. 

### 2.2. Data Collection and Pre-Processing

The principle of date selection was to select three pairs of Terra ASTER/MODIS LST images with each pair acquired in the same date. Considering the data availability and quality (e.g., low cloud cover), we carefully selected six Terra ASTER surface kinetic temperature scenes and three Terra MODIS LST datasets acquired on 04/03/2007, 4/28/2007, and 07/01/2007 at leaf-on seasons. Due to the scene coverage, two ASTER images acquiring in the same date had to be obtained and a mosaic built to fully cover the study area. All the images were acquired in April or July 2007 with no/low cloud cover. Planck’s Law was used to derive ASTER’s surface kinetic temperature based on the emissivity values from the Temperature-Emissivity Separation (TES) algorithm with ±1.5 K measurement error [45]. MODIS’s land surface temperature/emissivity was created using the generalized split-window LST algorithm with standard deviations of errors of 0.4–0.5 K [46]. Table 1 lists all the images, with their acquisition dates and spatial resolutions.

A study boundary was determined based on the overlap among three ASTER LST images acquired at different image dates. All images were then resized such that they comprised the same study boundary (Figure 1) without cloudy pixels (mainly near the coast or in the ocean). As a result, three ASTER/MODIS LST image pairs were ready for LST image fusion analysis. 

### 2.3. LST Image Fusion

The goal of LST image fusion in the study was to create simulated LST image at ASTER spatial resolution and MODIS acquisition dates. Since LST is associated to the energy exchange between the land surface and atmosphere [46], rather than a response to reflected energy, some traditional image fusion methods (e.g., IHS and PCA methods) may be not suitable for LST simulations. STARFM, a statistics-based approach was used to perform LST image fusion. Initially the model was developed to simulate 30-m surface reflectance images based on observed Landsat and MODIS surface reflectance images [16]. The model algorithm is given as:

(1) $$L\left(x_{\frac{\omega }{2}},y_{\frac{\omega }{2}},t_{0}\right)=\sum_{i=1}^{\omega }\sum_{j=1}^{\omega }\sum_{k=1}^{\omega }W_{ijk}\times (M\left(x_{i},y_{j},t_{0}\right)+L\left(x_{i},y_{j},t_{k}\right)-M\left(x_{i},y_{j},t_{k}\right))$$

where *L* represents the Landsat surface reflectance and *M* for MODIS, *ω* is the searching window size with ($x_{\frac{\omega }{2}},y_{\frac{\omega }{2}}$) as the central pixel, ($x_{i},y_{j}$) is a given pixel location for a Landsat and MODIS image pair, *t*_0_ is the acquisition date for a simulated date, and *t_k_* is the acquisition date for the image pair. *W_ijk_* is the weight deciding the influence of each neighboring pixel to the simulated reflectance of central pixel $\left(x_{\frac{\omega }{2}},y_{\frac{\omega }{2}}\right)$. Variable *W_ijk_* is defined by three components: spectral difference between Landsat and MODIS, temporal difference between the simulated and input MODIS images, and location distance between central pixel and candidate pixel [16]. The STARFM can accurately estimate the surface reflectance with pure MODIS pixels, and capture permanent land-cover changes during the growing season. However, fine-resolution bracketing (Landsat) images are necessary in capturing transient phenology for the STARFM [16]. The model is applicable to other instruments since its functioning is purely statistical in nature [16]. For example, one study utilized the STARFM model to produce interpolated ASTER surface reflectance images based on archived ASTER and MODIS surface reflectance images [4]. 

The STARFM model was adapted to simulate LSTs for the simulation date 04/28/2007 based on ASTER and MODIS LST images. More specifically, there were five input LST images: two ASTER and MODIS image pairs acquired on 04/03/2007 and 07/01/2007 respectively, and one MODIS LST image acquired on 04/28/2007. Since STARFM was designed to use Landsat and MODIS stimulated land surface reflectance as inputs, it was necessary to modify the model parameters (e.g., image size, spatial resolution, and maximum search distance) for use with ASTER and MODIS LST data. *W_ijk_* was determined based on three factors: LST difference between ASTER and MODIS imagery (an approximate calculation to identify the homogeneity of LST for a MODIS pixel), temporal changes on MODIS LST measurements between the simulation and the acquisition dates, and location distance between the central simulated pixel and the surrounding candidate pixel with similar LST associated. We adopted the assumptions made by Gao [16] when combining the three factors above for the calculation of *W_ijk_*. We naively assumed that: (1) homogeneous MODIS pixels provide identical temporal changes as ASTER observations in regard to LST values; (2) measurements with less change from the simulation date provide better reference for the prediction date; and (3) neighboring pixels with closer distance usually provide better reference for simulation. The LST image fusion was validated by statistically comparing the observed and simulated ASTER LSTs for date 04/28/2007. 

### 2.4. Downscaling Effect Analysis

The availability of LST or thermal images varies from location to location, so that it is important to assess the scaling effect, especially the downscaling effect of LST image fusion with STARFM. It is noted that downscaling in remote sensing refers to a decrease in pixel size and an increase in spatial resolution. The downscaling effect may associate with either consistent or inconsistent measurements in information retrieval [47]. In order to assess the possible downscaling effect, all the input images (both ASTER and MODIS LST images) were resampled to possess the following eight spatial resolutions (units: meters): 15, 30, 60, 90, 120, 250, 500, and 1000, based on the Cubic Convolution resampling method. It was noted that those scales range from 15 to 1000 m, so it seemed to be more appropriate to select a resampling approach that can generate a smooth instead of choppy output image, e.g., Cubic Convolution. A simulated ASTER-like LST image at a particular scale was generated by entering the corresponding image pairs at the same scale to STARFM. For example, 15 m LST image pairs (ASTER and MODIS) acquired at different dates (04/03/2007 and 07/01/2007) generated a 15 m simulated ASTER LST dataset for a particular date (04/28/2007). The basic statistics were calculated across the scales to identify the appropriate scales for LST simulation.

## 3. Results

### 3.1. Simulated ASTER LST Image

**Figure 2:**
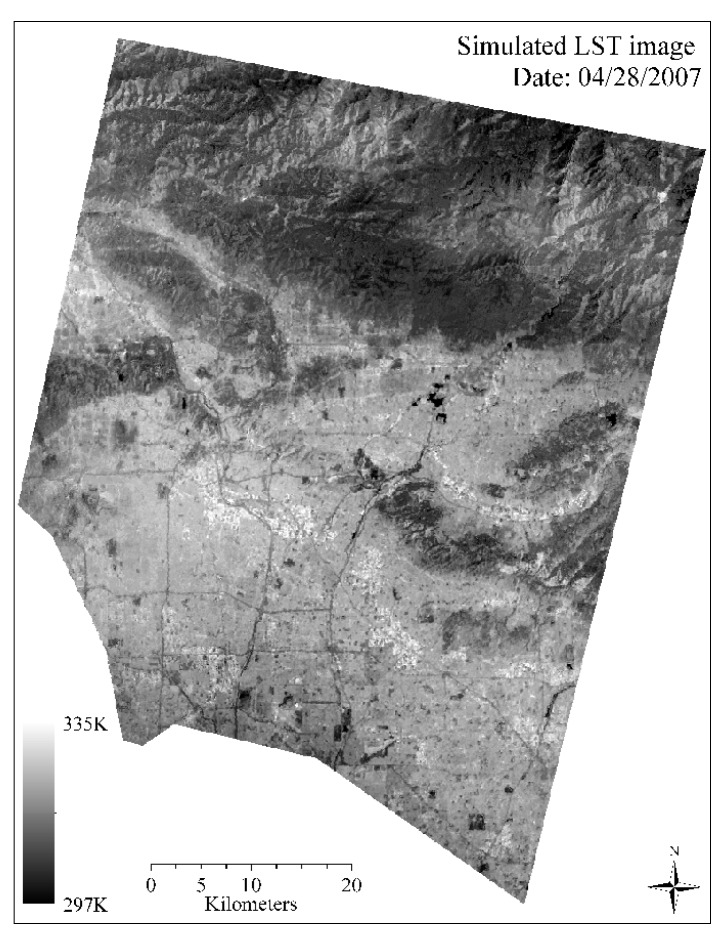
Simulated Advanced Spaceborne Thermal Emission and Reflection Radiometer (ASTER)-like land surface temperatures (LST) image on date 04/28/2007 (90 m spatial resolution).

Figure 2 shows the simulated ASTER LST image on date 04/28/2007 (90 m spatial resolution). As can be observed in the figure, overall the LSTs on the north side were lower than those in the south. The simulated LSTs tended to agree with land use and land cover types, and the variations of LSTs corresponded to energy balance across the surface. For example, LSTs in urbanized lands, e.g., south side with heavy urban infrastructures and buildings/houses possessed relatively higher LST values with lighter tones, while vegetated mountain areas on the north contained relatively lower LSTs with darker tones. LSTs along the major roads could be clearly observed at 90 m resolution. 

### 3.2. Image Fusion Validation

**Figure 3:**
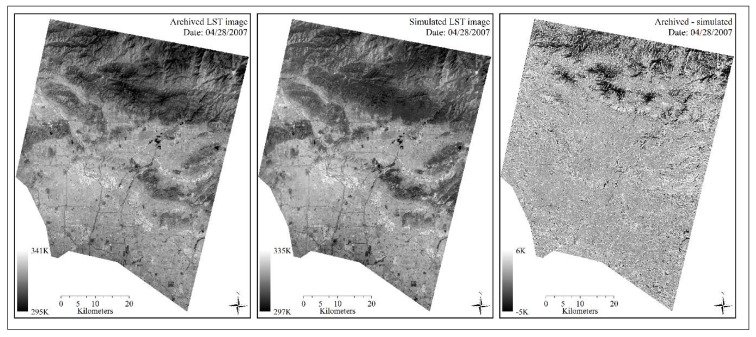
A comparison between observed (**left**) and simulated (**middle**) ASTER LST image on date 04/28/2007. The map (**right**) shows the difference between observed and simulated images. Spatial resolution: 90 m.

The observed ASTER LST and simulated ASTER LST images (date 04/28/2007, spatial resolution 90 m) had high agreement in terms of spatial variations and LST statistics (Figure 3 and Table 2). Urban impervious surfaces possessed much higher LSTs with lighter tones but mountainous areas showed dark tones with lower LSTs on both figures. It was notable that the simulated LST image appeared to contain slightly lower LST contrasts across the surface than those of observed LSTs. It indicated the possible influences of input ASTER and MODIS LST images on the fusion results. It is likely due to the calibrations of *W_ijk_* which were calculated based on LST difference between ASTER and MODIS imagery, temporal changes on MODIS LSTs between the simulation and the acquisition dates, and location distance between the central simulated pixel and the surrounding candidate pixel with similar LST associated. On the third panel (right) of Figure 3, the simulated LSTs of rural mountain areas on the north deviated more from observed LSTs at in situ pixels, comparing to urban pixels farther south. This finding may correspond to the limited capability of STARFM in the mountainous areas as addressed by Gao [16].

**Figure 4:**
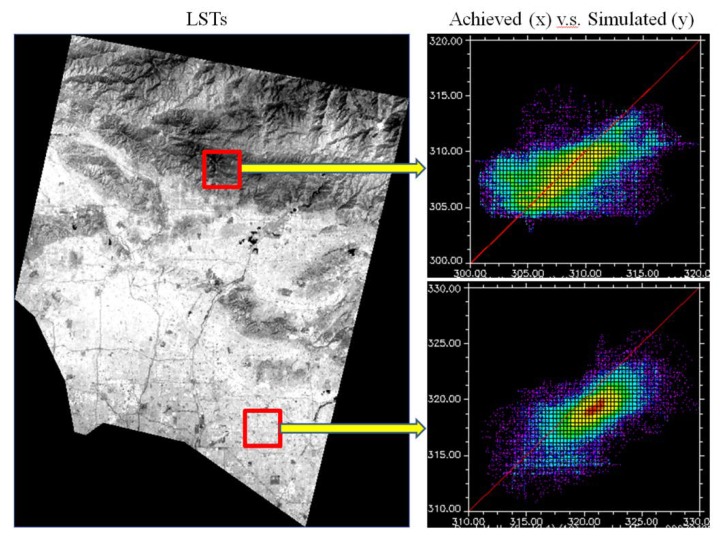
Scatter plots between observed and simulated ASTER LST datasets at mountain and urban areas on date 04/28/2007. Temperature units: K. Spatial resolution: 15 m.

In order to further demonstrate the variations of departures across the surface between observed and simulated images, a scatter plot was created to compare the observed and simulated LSTs in a rural mountain site, as well as a plot for an urban site (Figure 4). LSTs of mountain site tended to gather along the reference line in red with extensive departures on both sides of the line. It indicated that mountain LSTs generated larger errors in simulation (greater or smaller than observed LSTs). Urban LSTs also accumulated along the reference line with limited departures on the left side of the reference but much more on the right side. It implied that observed LSTs in the urban site had higher values than those of simulated LSTs. This difference could be once again associated with observed LST images used in STARFM. The statistics showed that the mean differences between observed and simulated ASTER LST datasets at 90 m spatial resolution reached 0.89 K, with a standard deviation (SD) of 1.93 by using Cubic Convolution resampling method (Table 2).

### 3.3. Downscaling Effect Analysis

**Figure 5:**
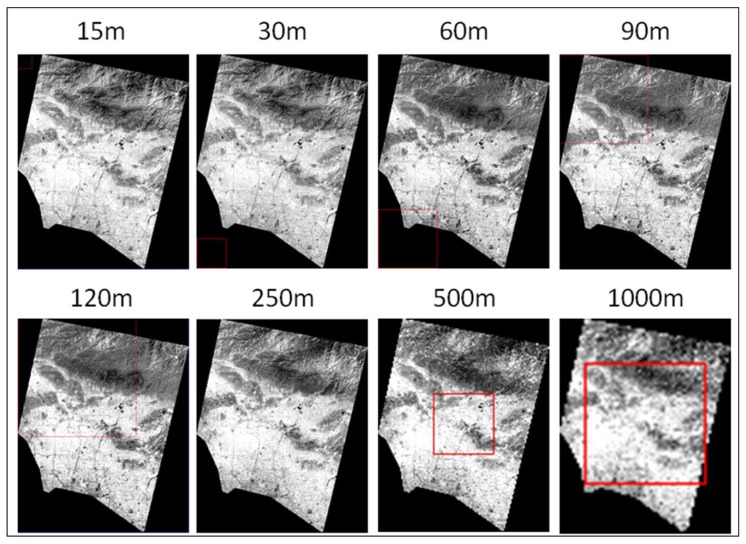
Simulated LST images across the scales.

Figure 5 shows a series of simulated ASTER LST images at different spatial resolutions: 15, 30, 60, 90, 120, 250, 500 and 1000 m. More variations could be observed when the scale changed from 15 to 120 m, and from 120 to 1000 m, LST distributions became more homogeneous. Based on the statistics (mean and SD) shown in Table 2, the mean LST differences were around 1 K from 15 to 1000 m resolution, except that at 120 m, which was much lower (−2.72 K). This exception implied the influence of input (MODIS and ASTER) LST images to the model. Overall there were slight increases across the scales in regard to SDs. However, SD was noticeably higher at 120 m (3.43), which corresponded to the low mean LST difference at this scale.

## 4. Discussion and Conclusions

This study simulated LSTs by using STARFM, an existing statistics-based image fusion model, and investigated the downscaling effect of LST fusion process based on ASTER and MODIS LST products. Results showed that LST image fusion reached a reasonable accuracy across the scales (15–1000 m) with both the Cubic Convolution and the Bilinear Convolution resampling methods. However, the results of the Nearest Neighbor resampling were not as consistent as those of the Cubic Convolution and the Bilinear Convolution methods. The downscaling process did not seem to significantly affect the fusion results, which suggested that the LST simulation approach was somewhat scale independent. Flat terrains yielded more accurate LST simulation than hilly and mountain areas. The results can be used in studies requiring LSTs with fine spatial details, e.g., time sensitive and heat-related epidemiological/public health studies, and monitoring the weekly/monthly shifts of urban heat islands (central locations and magnitudes) for the studied location. The low sensitivity to scaling effect makes it possible to apply the same approach to other urban locations. 

While the result of the simulation was promising, the demonstrated LST image fusion method should be used with caution. There are some potential limitations in adopting the STARFM for LST simulation. First, the accuracy of LST simulation was directly linked with the archived MODIS and ASTER LST products which may possess some errors introduced by LST retrieval algorithms [1]. Consequently the simulated LSTs could differ more from the LST measurements on the ground for some locations. This potential disagreement should be independent from the STARFM performance. 

Second, the temperature distribution across the land surface was obviously different from that of land surface reflectance since temperature variation was more closely related to the surface energy balance. In addition, the seasonal change of LST can be transient phenology. The variation of the surface energy balance may not be entirely accounted by such a statistics-based approach. STARFM could not generate accurate results on LST simulations without quality bracketing ASTER images. As such, more archived MODIS and LST images acquired at different dates and seasons would be helpful in calibrating the STARFM model and validating the results. 

Third, STARFM was believed to be less suitable for simulating spectral reflectance in the mountainous areas or heterogeneous landscapes with extreme surface reflectance, e.g., small agriculture patches, due to the fact that mixed coarse-resolution MODIS pixels usually captured limited variation in surface reflectance across the surface [16,18,19]. Future work may include a separation between flat terrain and hilly areas before performing LST simulation, and further optimization of STARFM parameters, e.g., *W_ijk_*, and maximum search distance. It would also be worthy to assess how LST varies with elevation by incorporating surface elevation as the topographic effect, as indicated by Wan and Dozier [48]. To provide even more details, an investigation of transitions could be conducted to demonstrate how simulated LSTs vary with land cover types, e.g., from highly developed downtown to residential areas with mixed vegetation and houses and mountains with low-to-median-density tree canopy. 

Meanwhile, it is worthwhile to compare the current LST simulation method with other image fusion models, such as STAARCH, in which spatial-temporal landscape changes can be better captured by choosing an optimal acquisition date for Landsat input image [18], ESTARFM that can better simulate the surface reflectance for complex and heterogeneous regions with the assistance of reflectance trend analysis and spectral unmixing approach [19], and SADFAT that incorporated annual temperature cycle modeling and spectral unmixing into the prediction of LST change [17]. 

The image resampling process could influence the results at certain levels. The Cubic Convolution method was first applied to create a series of LST images for downscaling effect analysis. In order to evaluate the possible influences of resampling approaches, two other traditional resampling methods, Bilinear Interpolation, and Nearest Neighbor method were also used to generate two sets of ASTER and MODIS LST images as inputs to STARFM. The same basic statistics were calculated based on those two resampling methods (Table 2). According to the statistics in Table 2, the Bilinear Interpolation method leaded to quite similar results to those of Cubic Convolution. However no surprise was found at 120 m resolution as that of Cubic Convolution, it might indicate that the input (resampled MODIS and ASTER) images had less influence on LST fusion at 120 m. The simulated LSTs with Nearest Neighbor method seemed to be similar as those of Cubic and Bilinear methods at 15–30 m resolution. However the simulated LST images tended to consistently depart from observed images at 60–1000 m with about −4.5 K mean difference and around 4.2 K standard deviation. The comparison between these three traditional resampling methods suggested that LST image fusion performs well with smooth resampling methods (e.g., Cubic and Bilinear) but reached less acceptable results with Nearest Neighbor method. 

It was notable that the study directly applied the traditional resampling approaches to upscale the ASTER LST imagery and meanwhile to downscale the MODIS LST imagery as inputs of STARFM model, rather than adopting the downscaling techniques used by other researchers, e.g., Zakšek and Ostir [36]. It will be worthwhile to apply other downscaling techniques and compare the results with current findings. 

## Figures and Tables

**Table 1:** Satellite images used in the study, their acquisition dates and spatial resolutions.

Satellite Data	Acquisition Date & Time (GWT)	Spatial Resolution (m)
Terra ASTER AST_08	3 April 2007, 18:46	90
(Surface Kinetic Temperature)	28 April 2007, 18:39 *	
	1 July 2007, 18:39	
Terra MODIS 11A1 LST/E	3 April 2007, daily	1000
(Land Surface Temperature & Emissivity)	28 April 2007, daily	
	1 July 2007, daily	

* image used for image fusion validation.

**Table 2:** Basic statistics in the difference maps between observed ASTER LST and simulated ASTER LST data (observed–simulated) for image date 04/28/2007, by using Cubic Convolution, Bilinear Interpolation, and Nearest Neighbor resample methods.

Spatial Resolution (units: m)	Mean (Units: K)			Standard Deviation (SD) (Units: K)		
	Cubic	Bilinear	Nearest Neighbor	Cubic	Bilinear	Nearest Neighbor
15	0.88	0.93	1.08	1.99	1.96	1.98
30	0.95	0.94	1.04	1.98	1.95	2.17
60	0.90	0.98	−4.59	1.94	1.95	4.25
90	0.89	0.88	−4.58	1.93	1.92	4.26
120	−2.73	0.89	−4.53	3.43	1.91	4.24
250	0.93	0.90	−4.45	2.14	1.88	4.09
500	0.90	0.86	−4.41	2.12	1.84	4.16
1000	0.92	0.90	−4.33	2.12	1.85	4.20
